# An overview of protective strategies against ischemia/reperfusion injury: The role of hyperbaric oxygen preconditioning

**DOI:** 10.1002/brb3.959

**Published:** 2018-03-30

**Authors:** Ciprian Hentia, Alex Rizzato, Enrico Camporesi, Zhongjin Yang, Danina M. Muntean, Dorel Săndesc, Gerardo Bosco

**Affiliations:** ^1^ Master II level in Hyperbaric Medicine Department of Biomedical Sciences University of Padova Padova Italy; ^2^ Faculty of Medicine “Victor Babeș” University of Medicine and Pharmacy Timișoara Romania; ^3^ TEAMHealth Research Institute TGH Tampa FL USA; ^4^ The Institute for Human Performance SUNY Upstate Medical University Syracuse NY USA; ^5^ Center for Translational Research and Systems Medicine “Victor Babeș” University of Medicine and Pharmacy Timișoara Romania

**Keywords:** hyperbaric oxygenation, ischemia–reperfusion injury, preconditioning

## Abstract

**Introduction:**

Ischemia/reperfusion (I/R) injury, such as myocardial infarction, stroke, and peripheral vascular disease, has been recognized as the most frequent causes of devastating disorders and death currently. Protective effect of various preconditioning stimuli, including hyperbaric oxygen (HBO), has been proposed in the management of I/R.

**Methods:**

In this study, we searched and reviewed up‐to‐date published papers to explore the pathophysiology of I/R injury and to understand the mechanisms underlying the protective effect of HBO as conditioning strategy.

**Results:**

Animal study and clinic observation support the notion that HBO therapy and conditioning provide beneficial effect against the deleterious effects of postischemic reperfusion. Several explanations have been proposed. The first likely mechanism may be that HBO counteracts hypoxia and reduces I/R injury by improving oxygen delivery to an area with diminished blood flow. Secondly, by reducing hypoxia–ischemia, HBO reduces all the pathological events as a consequence of hypoxia, including tissue edema, increased affective area permeability, postischemia derangement of tissue metabolism, and inflammation. Thirdly, HBO may directly affect cell apoptosis, signal transduction, and gene expression in those that are sensitive to oxygen or hypoxia. HBO provides a reservoir of oxygen at cellular level not only carried by blood, but also by diffusion from the interstitial tissue where it reaches high concentration that may last for several hours, improves endothelial function and rheology, and decreases local inflammation and edema.

**Conclusion:**

Evidence suggests the benefits of HBO when used as a preconditioning stimulus in the setting of I/R injury. Translating the beneficial effects of HBO into current practice requires, as for the “conditioning strategies”, a thorough consideration of risk factors, comorbidities, and comedications that could interfere with HBO‐related protection.

## 1 INTRODUCTION

Dramatic improvements in living conditions and health care have significantly increased human life expectancy by up to 40% over the past 50 years (worldbank.org). With the aging of the population, the incidence of pathologies associated with myocardial and cerebral ischemia is expected to increase, being largely favored by the fast‐rising pandemic of diabetes mellitus and obesity (Go et al., 2014). Importantly, ischemia–reperfusion (I/R) injury of both heart and brain shares common pathomechanisms represented by oxidative stress (Muntean et al., 2016; Sanderson, Reynolds, Kumar, Przyklenk, & Hüttemann, 2013), inflammation (Goldfine & Shoelson, 2017; Ong et al., 2018), microvascular dysfunction (Granger & Kvietys, 2017; Gursoy‐Ozdemir, Yemisci, & Dalkara, 2012), and, ultimately, cell death.

A great success has been achieved in reducing the ischemic injury, with the advent of revascularization procedures and the successful recanalization of the occluded arteries (Bhaskar, Stanwell, Cordato, Attial, & Levi, 2018) since past three decades. However, no treatment capable of mitigating the cell death occurring during the postischemic reperfusion is currently available in the daily practice (Heusch, 2017; Ibanez, Heusch, Ovize, & Van de Werf, 2015). A recent study shows that, although the mortality of heart attack decreased, the morbidity increased due to the development of heart failure (Hausenloy & Yellon, 2016). Reperfusion injury of the heart occurring most frequently in the setting of acute myocardial infarction and cardiac bypass surgery has been recently acknowledged as a “neglected therapeutic target” (Bulluck & Hausenloy, 2015; Hausenloy & Yellon, 2013).

Pathophysiology of myocardial I/R injury comprises reperfusion‐induced arrhythmias, myocardial stunning, microvascular obstruction, and lethal reperfusion injury (Bulluck & Hausenloy, 2015). Over the past 30 years, the quest for novel therapies able to protect myocardium against the deleterious effects of lethal reperfusion injury has lead to the identification of “ischemic conditioning” as the most powerful strategy of endogenous protection. The term refers to a series of brief episodes of ischemia alternated with reperfusions applied prior to or after a prolonged ischemia either locally (ischemic pre‐ and postconditioning) or at distance (remote ischemic pre‐ and postconditioning) that resulted in infarct size reduction in experimental setting and/or clinical outcome improvement in the clinical arena (reviewed by Heusch, 2015; Cohen & Downey, 2015; Hausenloy, 2013; Duicu, Angoulvant, & Muntean, 2013). A large body of research has aimed at characterizing the signal transduction of conditioning maneuvers in order to identify cellular/molecular targets that can be pharmacologically modulated (“pharmacological conditioning”). However, neither ischemic nor pharmacological conditioning strategies were translated so far into an effective protective therapeutic protocol in daily practice mainly due to various confounders such as comorbidities (e.g., diabetes and renal failure), several cotreatments, and aging (Bulluck & Hausenloy, 2015; Heusch, 2017).

Hyperbaric oxygen (HBO) has emerged more than a decade ago as putative protective pharmacological therapy in the setting of I/R injuries of brain and heart, in particular in the settings of ischemic stroke and acute myocardial infarction/revascularization procedures with encouraged outcomes. (Camporesi & Bosco, 2014; Francis & Baynosa, 2017; Yogaratnam et al., 2006).

In this study, we briefly review the pathophysiology of I/R injury and current treatment strategy. We further address the protective effects and mechanisms of in the treatment of I/R injury.

## 2 PATHOPHYSIOLOGY OF ISCHEMIA/REPERFUSION INJURY

Pathophysiology of myocardial I/R injury recognizes four types of specific lesions, namely reperfusion‐induced arrhythmias, myocardial stunning, microvascular obstruction, and the most severe lethal reperfusion injury. The intimate mechanisms responsible for the occurrence of these lesions are the direct results of I/R‐triggered changes in several cells that are briefly summarized in Table 1.

**Table 1:** Cellular changes occurring during I/R injury of the heart

	Cells	Pathophysiological changes
1.	Cardiomyocytes (Hausenloy & Yellon, 2016; Jennings, 2013; Kalogeris et al., 2017; Yellon & Hausenloy, 2007)	Ionic changes: intracellular calcium and protons accumulation (acidosis during ischemia and pH normalization at reperfusion) Impaired contractility: loss of contractile function (during ischemia) and hypercontracture (at reperfusion) Endoplasmic reticulum stress (with accumulation of misfolded/unfolded proteins responsible for the unfolded protein response that triggers both deleterious and protective signaling pathways at reperfusion) Mitochondrial changes: increased ROS generation and opening of the mitochondrial permeability transition pores (largely occurring at reperfusion) Cell death: via necrosis, apoptosis, autophagy, and regulated necrosis (e.g., necroptosis, ferroptosis, and pyroptosis)
2.	Cardiac fibroblasts (Ma, Iyer, Jung, Czubryt, & Lindsey, 2017; Turner & Porer, 2013; Valiente‐Alandi, Schafer, & Blaxall, 2016)	Transdifferentiation to myofibroblasts (with contractile and synthetic/secretory phenotypes responsible for both dynamic cardiac healing and remodeling with myocardial stiffness and progression to heart failure) Intercellular communication and cross talk with the extracellular matrix in the injured myocardium
3.	Endothelial and smooth muscle cells (Kalogeris et al., 2017; Korthuis, 2018; Turer & Hill, 2010)	Activation of endothelial cells that acquire a prothrombogenic phenotype (with the recruitment of inflammatory cells, myocardial infiltration, and damage) Increased vascular permeability (with subsequent microvascular dysfunction, edema formation, and increased interstitial fluid pressure) Impaired vasodilation (due to abnormal NO release, oxidative, nitrosative and nitrative stress, and adhesive endothelial cells–leukocytes interactions.)
4.	Pericytes (Bonaventura, Montecucco, & Dallegri, 2016; Jennings, 2013)	Ischemia‐induced contraction of microvessels with the aggravation of the capillary no‐reflow phenomenon (in the brain) Resolution of inflammation and stabilization of the scar (in the heart)
5.	Platelets (Barrabes, Mirabet, Agullo, Pizcueta, & Garcia‐Dorado, 2007; Gawaz, 2004)	Activation and aggregation (with platelet–leukocyte aggregation responsible for the aggravation of microvascular dysfunction and microembolization of the vascular bed)
6.	Immune cells (Bonaventura et al., 2016; Kalogeris et al., 2017; Prabhu & Frangogiannis, 2016)	Infiltration of the infarcted area with neutrophils and macrophages (accelerated at reperfusion and responsible for the activation of both pro‐inflammatory and anti‐inflammatory signaling pathways) Infiltration with T cells (T effector and Th1 cells with pro‐inflammatory effects and Th2 cells with protective effects) and B cells (with pro‐inflammatory properties). Attraction of dendritic cells (with T and B cells activation and infarct exacerbation in the brain, but mixed results in the heart)
7.	Mast cells (Kalogeris et al., 2017)	Activation and degranulation (with pro‐inflammatory effects, vascular leakage and interstitial edema exacerbation, neutrophil infiltration, and, in the brain, promotion of thrombolysis and hemorrhage)

Ischemia/reperfusion injury of the brain can be either focal as occurs in ischemic stroke which arises in a specific territory due to atherothrombotic or thromboembolic vascular occlusion (the most common clinical presentation) or global—in the setting of cardiac arrest followed by resuscitation and the neonatal hypoxic–ischemic encephalopathy (Sanderson et al., 2013). The mechanisms underlying cerebral injury at the postischemic reperfusion are similar to the ones triggering the above‐mentioned specific myocardial lesions, with a major contribution of mitochondria‐dependent oxidative stress (Sanderson et al., 2013). The brain exhibits a unique sensitivity to ischemia due to its highest metabolic activity, dependence on constant glucose delivery, and structural and functional particularities that render neurons more vulnerable to oxidative damage, namely increased polyunsaturated fatty acids in the cellular membranes and lower levels of antioxidant enzymes and mitochondrial cytochrome *c* oxidase as compared to the heart (Kalogeris, Baines, Krenz, & Korthuis, 2017).

## 3 THE SAGA OF CONDITIONING STRATEGIES

In the setting of acute I/R injury, the most powerful cardioprotective strategy, apart from revascularization, is the so‐called ischemic preconditioning (IPC). The term was coined by the group of Robert Jennings which firstly reported that four episodes of nonlethal ischemia applied prior to the onset of a prolonged lethal episode (index ischemia) dramatically reduced (by 75%) the size of experimental myocardial infarction in dogs (Murry, Jennings, & Reimer, 1986). After the first “wave of doubt” that additional ischemia could paradoxically be beneficial, several research groups confirmed the protective effects of IPC in different experimental models of cardiac I/R injury in all animal species: dog (Gross & Auchampach, 1992; Murry et al., 1986), pig (Schott, Rohmann, Braun, & Schaper, 1990), rabbit (Toombs, Wiltse, & Shebuski, 1993), rat (Yellon, Alkhulaifi, Browne, & Pugsley, 1992), and monkey (Yang et al., 2010). Protection elicited by IPC appears immediately after a brief I/R period and lasts for a few hours.

A few years after the initial observations were made, a similar protection was observed and described which appears after a I/R injury and lasts for a couple of days. It was described as “late preconditioning” or “the second window of protection”, and the earlier one is acknowledged as “early preconditioning” or “the first window of protection” (Kuzuya et al., 1993). The early phase (first window of protection or “early or classic preconditioning”) which is initiated within minutes after the preconditioning stimulus provides strong anti‐infarct protection but lasts for only a few hours. After approximately 12 hr of no apparent protection, a late phase (second window of protection or “late or delayed preconditioning”) occurs and provides a longer (albeit less robust) protection lasting for 3 to 4 days. The mechanisms underlying these phases are different; the early protection is provided by rapid modifications of the existing structures, while the late protection occurs later because it requires the activation of specific genes and de novo synthesis of proteins (Berger, Macholz, Mairbäurl, & Bärtsch, 2015).

Przyklenk, Bauer, Ovize, Kloner, and Whittaker (1993) reported that IPC‐related protection was also provided to the remote “virgin” myocardium, meaning that the mediators that signal cardioprotection are capable to leave the ischemic cells and act on the nearby structures. Furthermore, it has been discovered that these protective molecules apparently are also released into the blood and thus are able to transfer protection to other organs. For example, an episode of renal ischemia confers protection to the myocardium in rats (Gho, Schoemaker, van den Doel, Duncker, & Verdouw, 1996) and transient ischemia of a limb provides cardioprotection similar to that induced by classic IPC (Birnbaum, Hale, & Kloner, 1997). This phenomenon was denominated “remote ischemic conditioning” (RIC) and has been intensively studied over the past decade due to its high translational potential in the clinical arena. RIC is a noninvasive, easily applicable, and inexpensive preconditioning strategy. Recently, researchers have discovered that RIC can be triggered by mechanical, chemical, and electrical stimuli, and the protective signal is transferred by both humoral and neuronal pathways (Heusch, 2015). RIC provides protection basically to all organs that may be subjected to I/R injury: brain, kidney, liver, intestine, stomach, lung, skeletal muscle, etc. (Candilio, Malik, & Hausenloy, 2013). However, all the large trials that investigated the benefits of RIC in the setting of cardiac surgery or percutaneous coronary intervention had disappointing results so far (Hausenloy & Yellon, 2016). This may be due to either confounding factors such as comorbidities/comedications or, in some cases, a fault study design (Heusch, 2017). Also, the anesthetic regimens during the interventions may have interfered with the expected results (Zaugg & Lucchinetti, 2015). In this respect, the novel (combined) cardioprotective therapies should be investigated in multitherapy models (Bell et al., 2016; Hausenloy et al., 2017).

## 4 THE SIGNAL TRANSDUCTION OF IPC

### 4.1 The triggers

The IPC triggers are stimuli that act during the brief ischemic episode, activate the signal transduction pathways in a receptor/nonreceptor manner, and transmit the protective signal to the *effector(s)* through *mediators* (Downey, Krieg, & Cohen, 2008). Some trigger molecules (adenosine, bradykinin, opioids, natriuretic peptides, and other cytokines) released during the conditioning IPC episodes activate the signaling cascades through specific membrane receptors. Other triggers, such as reactive oxygen species (ROS) and nitric oxide (NO), initiate the signaling cascades in a receptor‐independent manner (Heusch, 2008). Inside the cell, cytosolic signal transducers interact at different levels and at different time points (before lethal ischemia or at reperfusion) to convey information to the end effectors: mitochondria, the main organelles that ultimately control cell death in the setting of I/R injury (Cohen & Downey, 2015).

Adenosine, bradykinin, and opioids are triggers that act on G protein‐coupled receptors which, in turn, activate protein kinase C (PKC). Although the pathways are slightly different, they all converge on the PKC, the blockade of which results in the lack of any possible protection attributable to those triggers (Cohen & Downey, 2015). At variance, both exogenous (Nakano, Liu, Heusch, Downey, & Cohen, 2000) and endogenous (Cohen, Yang, & Downey, 2006; Krieg et al., 2009) NO can trigger myocardial protection in a receptor‐independent manner; in this case, protection occurs either dependent or independent of the activation of protein kinase G (PKG) signaling pathway (Sun et al., 2013). The next step identified within the IPC signal transduction consisted in the activation (opening) of the ATP‐sensitive K^+^ channel (K_ATP_) at the inner mitochondrial membrane (Garlid et al., 1997; Gross & Auchampach, 1992; Liu, Sato, O'Rourke, & Marban, 1998). The opening of mitochondrial K_ATP_ is related to electrochemical changes in the mitochondrial matrix that are responsible for an increased ROS production reported to occur mainly (but not exclusively) at the postischemic reperfusion. ROS can directly activate the PKC isoforms whose contribution to protection is species‐dependent, with PKCε being responsible for protection in the rodent heart, PKCα in large mammals, whereas controversial data are available about PKC (Cohen & Downey, 2015; Heusch, 2015). Activated PKC phosphorylates several downstream targets, among which connexin 43 (Cx43) plays a critical role in transferring the protective signal to mitochondria (recently reviewed by Boengler & Schulz, 2017).

One of the most important discoveries with respect to the preconditioning‐related cardioprotection is that minute ROS generation during the brief reperfusions is mandatory for IPC‐related protection, as ROS scavenging blocked protection; moreover, protection was lost when a hypoxic solution was used for reperfusion during the preconditioning phase (Dost, Cohen, & Downey, 2008). Importantly, the identification of the ROS sources and the threshold at which ROS loses potentially protective effect and become damaging to cellular function and integrity is still unclear in the field of cardioprotection (Di Lisa et al., 2011).

### 4.2 The mediators

The above‐described triggers act as stimuli to activate a couple of cytosolic enzymatic cascades that act as “mediators” during the index ischemia and/or at reperfusion in order to transmit the cardioprotective signal onto the final “effector(s)” that ultimately are responsible for the attenuation of the irreversible injury during the postischemic reperfusion.

By far, the most investigated signaling cascade activated during the early reperfusion following the index ischemia is represented by so‐called reperfusion injury salvage kinases (RISK) pathway (Hausenloy & Yellon, 2004). The RISK pathway comprises phosphoinositide 3‐kinase (PI3K), protein kinase B (Akt), and extracellular signal‐regulated kinase (ERK), which are proven effective in protecting the myocardium in rat (Hausenloy, Tsang, Mocanu, & Yellon, 2005) and rabbit (Yang et al., 2004). They act on endothelial nitric oxide synthase (eNOS) directly and on glycogen synthase kinase 3 beta (GSK3β) through one ribosomal protein kinase, P70S6K (Kleinbongard & Heusch, 2015).

Another signaling pathway is the survivor activating factor enhancement (SAFE) pathway (Lacerda, Somers, Opie, & Lecour, 2009; Lecour, 2009). At reperfusion, possibly due to the inflammatory response, the tumor necrosis factor (TNF) activates the Janus kinase (JAK) (a tyrosine kinase associated with the membrane receptor; it has a major role in translating signals from the cytosol to the nucleus) and signal transducers and activators of transcription (STATs) (when phosphorylated by the activated JAK, these dimerize and translocate to the nucleus, resulting in gene transcription; they may also be phosphorylated directly by receptor tyrosine kinases such as epidermal growth factor receptor, or by nonreceptor tyrosine kinases such as Src), playing an important role on the expression of the stress‐responsive genes (Willis, Homeister, & Stone, 2014). The effects on I/R happen far too quickly to be explained only by the gene transcription. It seems that STAT also phosphorylates GSK3β, inactivating it (Lacerda et al., 2009). Isoform STAT3, shown to be present in mitochondria, may also act on cyclophilin D, the target for mitochondrial permeability transition pore (mPTP) inhibitor cyclosporin A, thus inhibiting pore opening. Other downstream targets of STAT include proteins involved in cell survival and proliferation (Bcl‐2, Bcl‐xl, Mcl‐1, and p21) and growth factors (vascular endothelial growth factor) (Brantley & Benveniste, 2008). It also inactivates the proapoptotic factor Bad. TNF‐α's effect is concentration‐dependent, and high doses may increase the infarct size (Lecour, 2009). The SAFE pathway may also be activated by triggers other than TNF‐α via STAT: opioids, insulin, and sphingosine‐1 (Willis et al., 2014).

### 4.3 The effectors

The end effector of preconditioning through these signaling pathways, which interact with each other at different levels and different time points, is the mPTP, a protein structure—the structure of which is still controversial—located in the inner mitochondrial membrane. Inhibition of this high‐conductance pore is considered to be the final step in the protective signal transduction (Griffiths & Halestrap, 1993, 1995; Hausenloy, Maddock, Baxter, & Yellon, 2002; Hausenloy, Ong, & Yellon, 2009). When open, this pore dissipates the transmembrane electrochemical gradient used for ATP generation, resulting in ATP depletion, enhanced ROS production, the failure of energy‐driven membrane ion pumps, solute entry, organelle swelling, and, finally, mitochondrial rupture. The acidosis during the ischemic phase inhibits the formation of the pore. But during the reperfusion phase, the formation of the pore is stimulated due to alkalization of the pH, increasing mitochondrial Ca^2+^, and ROS (Cohen & Downey, 2015).

All cardioprotective signaling pathways inhibit the mPTP from opening. Both the RISK and SAFE cascades appear to have a final kinase, GSK3β, which seems to act differently to the other kinases, GSK3β being essential in pore formation. Conditioning signals lead to the inhibition, not activation, of this kinase, thus blocking mPTP formation and opening (Gross, Hsu, & Gross, 2004; Juhaszova et al., 2004; Tong, Imahashi, Steenbergen, & Murphy, 2002). Pharmacological activation of P70S6K leads to phosphorylation and inhibition of GSK3β, which further inhibits mPTP formation and opening, mimicking ischemic conditioning (Förster et al., 2006).

## 5 HYPERBARIC OXYGEN THERAPY

Hyperbaric oxygen (HBO) refers to the administration of 100% oxygen at two to three times the atmospheric pressure at sea level. HBO is a therapeutic strategy aimed at raising the arterial oxygen tension and the oxygen supply via an increase in oxygen dissolved in plasma that, ultimately, drives cellular respiration and sustains ATP synthesis in ischemic/hypoxic tissues. Over the time, HBO has been proven to be beneficial in acute conditions associated with general hypoxia/anoxia, such as carbon monoxide poisoning, circulatory arrest, and local ischemia/hypoxia, that is, cerebral and myocardial ischemia.

The systematic investigation of HBO as therapeutic measures during or after an ischemic insult of the brain and heart can be traced back to pioneering studies of George Smith (Smith, 1964; Smith & Lawson, 1963; Smith, Lawson, Renfrew, Ledingham, & Sharp, 1961). Indeed, this author firstly reported the preservation of cortical electrical activity in an experimental model of cerebral ischemia in the presence of compressed oxygen (Smith, 1964; Smith & Lawson, 1963; Smith et al., 1961). As for the heart, he reported in the in vivo model of regional I/R injury in dogs a significant decrease in mortality by preventing the occurrence of ventricular fibrillation in animals that breathed oxygen at two atmospheres absolute as compared to the groups that breathed room air or oxygen at one atmosphere absolute (Smith, 1964). A plethora of experimental studies further confirmed the HBO‐related neuroprotective effects and improved survival in animal models of middle cerebral artery occlusion, especially when applied at 2.0 absolute atmospheres (ATA) immediately after occlusion and for more than 6 hr (Xu et al., 2016). By facilitating oxygen delivery, HBO ameliorated cerebral circulation, decreased cerebral edema, blocked inflammatory cascades, and ultimately reduced infarct size via the mitigation of cell death and the restoration of mitochondrial oxidative phosphorylation (Sanchez, 2013).

In the coming years, several proof‐of‐concept clinical studies have been carried out to confirm the beneficial effect of HBO in the setting of brain ischemia associated with stroke with both positive results (several applications of HBO at 1.5 to 2 atmospheres absolute (ATA)—Neubauer & End, 1980) and neutral results (Nighoghossian, Trouillas, Adeleine, & Salord, 1995; Rusyniak et al., 2003). However, the current opinion is that the reduced number of randomized, double‐blind controlled trials does not provide enough evidence‐based decisions for the design of appropriate clinical protocols (Zhai, Sun, Yu, & Chen, 2016). Indeed, in the most recent meta‐analysis, seven of the 11 randomized trials showed no significant difference observed in the mortality rates at 6 months in the HBO‐treated patients as compared with the nontreated ones. However, these authors did not exclude the potential clinical benefit of the therapy as they found an improvement in a couple of disability and neurological function scale scores with HBO therapy (Bennett et al., 2014). Clearly, future randomized clinical trial will shed light on the benefits of HBO application together with thrombolysis within the same therapeutic window of 3 to 6 hr in acute stroke as well as in the poststroke stage in stable patients via the modulation of neuroplasticity.

## 6 HBO PRECONDITIONING (HBO‐PC)

Despite the fact that the beneficial effects of high‐pressure oxygen delivery in the setting of I/R injury of brain and heart were investigated in experimental and clinical settings for more than half a century and three decades before the discovery of the IPC phenomenon by the group of Robert Jennings (Murry et al., 1986), a search of medical databases for “hyperbaric oxygen preconditioning” returns a little over 100 articles (133 on PubMed to date), while searching for “ischemic preconditioning” returns nearly 10,000 articles (10,030 on PubMed to date).

Wada et al. (1996) was the first to use a protocol of repeated HBO (five sessions every other day) as compared to one single session prior to an episode of 5 min of forebrain ischemia elicited by the occlusion of both common carotid arteries and reported an increased tolerance against ischemic neuronal damage via the induction of HSP‐72 synthesis.

In 2000, Xiong recapitulated the beneficial effects of repeated HBO exposure on the induction of ischemic tolerance against focal cerebral ischemia (2000). Of note, the repeated cycles of HBO induced tolerance against transient ischemia (2 hr occlusion of the middle cerebral artery) but not against permanent ischemia (definitive occlusion). As in the case of IPC, tolerance was “dose‐dependent”; five cycles of HBO offered more than double protection as compared to three cycles of HBO with a reduction in the mean infarct size from 40.6 to 16.2 mm^3^. The discussion was whether the protection relates to the oxygen preload or is something similar to other forms of preconditioning already demonstrated. It has already been reported that reperfusion after prolonged ischemia had more deleterious effects than the ischemia itself, so scientists attempted to investigate how oxygen could protect against future oxygen damage, and they focused on ROS generation. In 2001, an article was published showing that HBO pretreatment conditioned the heart by enhancing enzymatic activity and gene expression of catalase, an important antioxidant enzyme; the protection was completely abolished in the presence of a catalase inhibitor (Kim et al., 2001).

**Figure 1:**
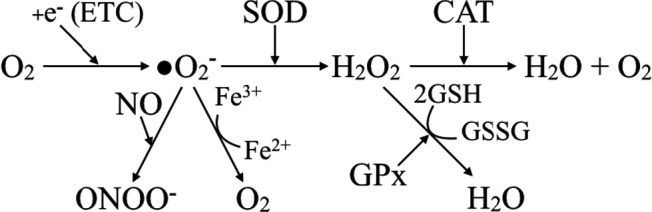
ROS formation and neutralization. (etc.—electron transport chain, NO—nitric oxide SOD—superoxide dismutase, GPx—glutathione peroxidase, and ONOO—peroxynitrite anion)

The generation and scavenging of ROS are depicted in Figure 1. A minor fraction (less than 1%) of the electrons flowing through the electron transport chain reacts with O_2_ to form superoxide, a highly reactive oxidant, which is converted by superoxide dismutase (SOD) into a less toxic molecule, H_2_O_2_. This is further converted by catalase into H_2_O and O_2_ or by glutathione peroxidase into H_2_O, the second reaction requiring the glutathione system. It may also transfer its electron, generating other oxidizing agents such as peroxynitrite (ONOO^−^). Other antioxidants such as lactoferrin, an iron‐binding protein, convert superoxide to oxygen.

Two important issues raised by the researchers were as follows: i) which of the two components of HBO (hyperoxia or hyperbaricity) act to induce the tolerance against I/R injury and ii) how does it act to accomplish this? In a model of spinal cord ischemia in rabbits, HBO pretreatment (2.5 atmospheres absolute [ATA], 100% O_2_) induced ischemic tolerance in the spinal cord in terms of both histopathology and motor function, but simple hyperbaricity (2.5 ATA, 21% O_2_) did not (Dong et al., 2002). The same experiments showed that normobaric exposure at 1 ATA, 100% O_2_ was associated with better histopathological outcome compared to the control group. Thus, hyperoxia appears to be the acting component. One might question whether the results were a matter of oxygen preload, but the researchers designed the study in a way to minimize this possibility: the index ischemia was provoked 24 hr after the last oxygen administration (Dong et al., 2002).

Another similar study (Nie et al., 2006) compared the effects of HBO on ischemic tolerance in rabbits. SOD and catalase activities were significantly higher with HBO treatment, while no difference between the hyperbaric air and control groups was found. The addition of a catalase inhibitor diminished the favorable increases in SOD and catalase activities. Moreover, by administering a potent free radical scavenger (dimethylthiourea) before HBO treatment, the increase in antioxidant activity was completely abolished. It was concluded that HBO increases ROS formation that triggers signaling pathways to finally upregulate antioxidant enzymes which protect from I/R injury. Similar results were obtained by other researchers (Cui et al., 2008; Li et al., 2008). With studies on ischemic preconditioning demonstrating the favorable role of ROS as triggers/mediators in the signaling pathways of protection, scientists began to dissect the similarities between the two ways of protection, questioning whether HBO might become “the magic bullet” of cellular protection (Yogaratnam et al., 2006).

Li, Li, Zhang, Wang, and Xiong (2007) studied the effects of HBO on cultured cells subjected to oxidative insult by H_2_O_2_ that caused severe DNA damage and decreased overall function and viability. The protective effects started 4 hr after the treatment and lasted for at least 24 hr. An increase in the inducible form of heme oxygenase (HO‐1) was reported in treated cells; when applying a specific HO‐1 blocker before the HBO treatment, protection was abolished. This finding supported the hypothesis that upregulation of HO‐1 plays an essential role in HBO preconditioning. Upregulation of HO‐1 by HBO was also demonstrated by other studies (Feng et al., 2015; He et al., 2011; Liu, Sun, Liu, Kang, & Deng, 2011), while the inhibition of enzyme activity with zinc protoporphyrin IX abolished the protective effects (Feng et al., 2015; Liu et al., 2011).

Gu, Kehl et al. (2008); Gu, Li et al. (2008) studied the hypoxia‐inducible factor‐1α (HIF‐1α) and its target gene erythropoietin (EPO). The transcription factor HIF‐1 is responsible for the induction of genes that facilitate survival under hypoxic conditions (Semenza, 1998). It consists of two subunits, the HIF‐1α and HIF‐1β (Wang, Jiang, Rue, & Semenza, 1995). HIF‐1α is an oxygen‐sensitive subunit and is expressed during hypoxic conditions, while HIF‐1β is constitutively expressed. Under normal conditions, HIF‐1α undergoes quick degradation (half‐life of 5 min), but in hypoxic conditions, its structure and transactivation are regulated by a series of signaling pathways (Masoud & Li, 2015). HIF‐1 appears to be of great importance in metabolic control and adaptation, which finally result in ischemic tolerance (Bergeron et al., 2000; Bernaudin et al., 2002) and cross‐tolerance (Maloyan et al., 2005; Shein, Horowitz, Alexandrovich, Tsenter, & Shohami, 2005).

There are more than 100 downstream genes involved in glucose metabolism (glycolysis pathway and glucose transporters), cell proliferation (TGF‐β3, EGF, and EPO), migration, and angiogenesis (vascular endothelial growth factor). Because it had been recently discovered that increased ROS levels may upregulate HIF‐1 expression (Kietzmann & Gorlach, 2005; Peng et al., 2006), and there had been evidence that EPO may exert potent neuroprotective effects (Morishita, Masuda, Nagao, Yasuda, & Sasaki, 1997; Sakanaka et al., 1998), the researchers studied in parallel the clinical, histological, and molecular effects of HBO preconditioning in a rat focal cerebral ischemic model (Gu, Kehl et al. 2008; Gu, Li et al. 2008). After HBO treatment (or normobaric normoxia, in the control group), the focal cerebral ischemia was obtained by injecting endothelin into the middle cerebral artery. The HBO group showed superior functional recovery and significantly decreased infarct size. A significant increase in HIF‐1α and EPO levels in the brain was also reported. HIF‐1α DNA‐binding activity was also increased, associated with an increase in the expression of downstream target genes (only mRNA expression of EPO was measured and showed significantly higher compared to controls). The researchers concluded that HBO preconditioning increased HIF‐1α DNA‐binding activity and the mRNA expression of EPO, a downstream gene of HIF‐1, followed by the increased protein expressions of HIF‐1α and EPO. They also offered an interesting discussion on EPO and its pathways: EPO–EPOReceptor–JAK‐2–PI3K, mitogen‐activated protein kinase (MAPK), STAT5, and common signaling pathways of ischemic and HBO conditioning (Gu, Kehl et al. 2008; Gu, Li et al. 2008). The HBO preconditioning through the HIF‐1 pathway was verified in other models: global hypoxia in mice (Peng et al., 2008) and rat liver ischemia (Ren et al., 2008).

Li et al. (2009) used HBO to obtain an apoptotic inhibition via the mitochondrial pathway, and the findings were similar to that of SAFE activation: reduced cytochrome C levels, decreased caspase‐3 and caspase‐9 activity, and increased Bcl‐2 and Bax proteins. Yamashita et al. (2009) discovered that HBO suppresses the p38 MAPK (a MAPK involved in cell differentiation, apoptosis, and autophagy), conferring the same protection as a p38 inhibitor. Qin et al. (2007) reported similar findings on the p44/42 MAPK, the activation of which by HBO was followed by protection, which was abolished by an activation inhibitor.

Furthermore, scientists addressed the dynamics of NO after HBO treatment. The first article to show the relation HBO ‐ NO ‐ protection was published in 2008 (Yogaratnam et al., 2008), which demonstrated that HBO stimulates the endogenous production of NO, which reduces neutrophil sequestration and adhesion and improves vascular flow. At the same time, Liu et al. (2008) found that HBO stimulates the mRNA of both eNOS and neuronal NOS to increase the NO levels. These were favorable to ischemic tolerance, but were associated with increased sensitivity to convulsions and seizures during subsequent oxygen exposures, probably through increased substrate for peroxynitrite formation. Wang et al. (2009) showed that HBO preconditioning had favorable effects in a model of spinal cord ischemia in rats by enhancing the activities of SOD, catalase, and Bcl‐2 expression in the mitochondria; in parallel, cytosolic cytochrome C was reduced and subsequently attenuated the activity of caspase‐9 and caspase‐3 (responsible for apoptosis). They also found increased NO production in the HBO group, and using a nonselective NOS synthase inhibitor (L‐NAME), the benefits of HBO preconditioning were abolished, demonstrating the importance of NO in the signaling pathway.

**Figure 2:**
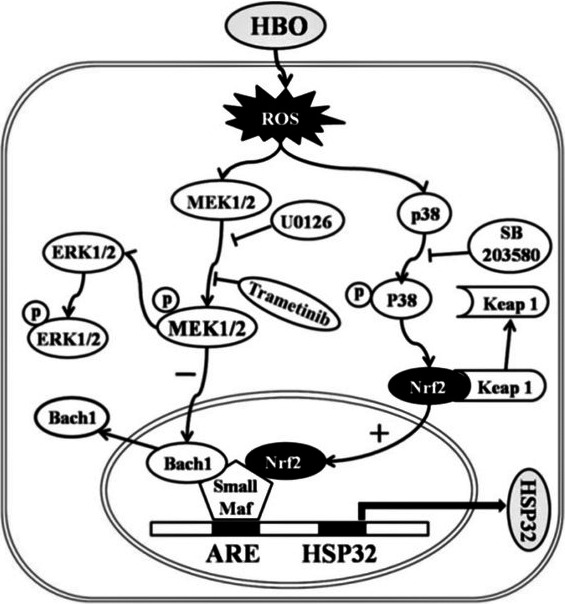
Signaling pathways triggered by HBO exposure and HSP32 expression in rat spinal neurons (from Huang et al., 2016)

Very recently, Huang et al. (2016) investigated the molecular mechanisms involved in HBO preconditioning and its complex relations with chemical mediators released in cell cultures from rat spinal neurons. They observed increased intracellular levels of ROS and NO after HBO preconditioning, and also the lack of these modifications when N‐acetyl‐L‐cysteine (NAC, a ROS scavenger) or L‐NAME were used prior to HBO treatment. To determine whether there was a cross talk between the two pathways, they studied the effects of ROS scavenger on NO production and the effects of NOS inhibitor on ROS generation. Neither had any effect on the other's production and generation. They further investigated the expression of HO‐1 or heat‐shock protein 32 (HSP32), as mentioned in the article (Figure 2).

The Keap1–Nrf2 pathway is the major activator of cytoprotective responses to endogenous and exogenous stresses caused by ROS (Kansanen, Jyrkkänen, & Levonen, 2012). HBO increases intracellular ROS formation, which activates both MEK1/2 and p38 MAPK. The activation of p38 MAPK initiates the transcription of the HSP32 gene. At the same time, the activation of MEK1/2 inhibits Bach1 disassociation from small microphage‐activating factor proteins, which prevents the surge of HSP32 gene transcription.

In the experiment, the expression of HSP32 was significantly inhibited by p38 MAPK blocker (SB203580) or Nrf2 gene knockdown, but was significantly enhanced by ERK1/2 inhibitor. The researchers concluded that HBO upregulates HSP32 by p38 MAPK and Nrf2 activation, and ERK1/2 may be negative regulators in this process. NAC significantly inhibited the activation of MEK1/2, p38 MAPK, and Nrf2 after HBO preconditioning, which demonstrated the role of ROS in this process. Using specific blockers for MEK1/2 (U0126) and p38 MAPK (SB203580), it was determined that only p38 MAPK acts on Nrf2. Because no blocker had any effect on the other target, they concluded that there was no cross talk between the two pathways.

Another recent study from Yin et al. (2015) demonstrated the role of the PI3k/Akt/Nrf2 protective mechanism in HBO preconditioning. In a model of I/R with in situ mice hearts, in the group treated with hyperbaric oxygen, these authors observed—apart from hemodynamic and histological advantages—an increased expression of HO‐1 (or HSP32), of Nrf2, and of Akt activity. By blocking PI3K, HO‐1 increase and cardioprotection were lost. Also, Nrf2 knockout or blocking Akt abolished the protective mechanisms.

Although many different studies replicated those results and found other proteins that play a role in the mechanism of HBO preconditioning, a full understanding of the mechanisms has not yet been presented.

## 7 OTHER BENEFITS OF HBO

Apart from these molecular findings, studies have also shown histological modifications after HBO treatment. HBO pretreatment reduced postoperative cerebral edema and improved neurological outcomes after surgical brain injury in mice (Jadhav et al., 2010). HBO also appeared to be neuroprotective against optical nerve insult via inhibition of neuronal apoptosis pathways in a rat model (Wang, Xu et al., 2010). HBO‐induced autophagy in the case of cerebral I/R injury was also found to be neuroprotective (Wang, Zhang, Du, Wang, & Sun, 2010). HBO exposure correlated with the increased expression of many protective genes (while normobaric oxygen did not), which resulted in stimulated protection and repair of the microvascular endothelial cells (Godman et al., 2010).

## 8 CLINICAL TRANSLATION OF EXPERIMENTAL FINDINGS

There is no doubt that both ischemic and pharmacologic (including HBO) preconditioning are powerful protective methods. They share most of the pathways of signal transduction from the conditioning stimulus to the final effector. Their benefit depends on factors regarding both the patient and the method.

Studies conducted on young healthy animals showed a great protective effect from conditioning protocols. Some randomized clinical trials have shown the efficacy of those strategies in humans to be not significant (Abdelnoor, Sandven, Limalanathan, & Eritsland, 2014; Brevoord et al., 2012). There are many differences between the populations of the experimental and clinical trials: animals were often young, derived from inbred strains, of the same age, and in good health; while the humans were typically old, with serious comorbidities for which they were taking various medications.

Several *comorbidities* were reported to interfere with the preconditioning‐related cardioprotection. Hypercholesterolemia (Ferdinandy, Szilvassy, & Baxter, 1998) impairs NO synthesis and peroxynitrite clearance (Kocsis et al., 2010), inhibits HSP70 regulation (Csont et al., 2002), and activates caspase‐3 (Wang et al., 2002). Diabetes alters the phosphorylation of PI3K‐Akt, decreases the generation of NO and eNOS (Gu, Kehl et al. 2008; Gu, Li et al. 2008), and generates abnormal ERK1/2 activity (Rana et al., 2015), K_ATP_ dysfunction (del Valle, Lascano, & Negroni, 2002), and activation of GKS‐3β (Yadav, Singh, & Sharma, 2010). Hypertension is the first comorbidity in patients with acute myocardial infarction or stroke (Go et al., 2014; Wagner, Ebner, Tillack, Strasser, & Weinbrenner, 2013). Hypertension is responsible for cardiac hypertrophy and oxygen imbalance, and clinical studies have shown the loss of preconditioning (Lorgis et al., 2012) probably through reduced Akt and GSK3β phosphorylation. Obesity *per se* engenders increased mitochondrial oxidative stress and impaired activation of mitochondrial K_ATP_ (Katakam et al., 2007). Aging engenders a reduction in norepinephrine and α‐adrenergic receptor activation (Abete et al., 1996), reduced translocation of PKC (Tani, Honma, Hasegawa, & Tamaki, 2001), a decrease in ERK phosphorylation (Przyklenk, Maynard, Darling, & Whittaker, 2008), and STAT‐3 deficiency (Boengler et al., 2008).

Furthermore, some of the common medications used were found to have effects on pharmacological preconditioning: nicorandil (Sakai, Yamagata, Teragawa, Matsuura, & Chayama, 2002), sildenafil (Kukreja et al., 2005), erythropoietin (Baker, 2005), opiates (Murphy, Szokol, Marymont, Avram, & Vender, 2006), cyclosporine (Piot et al., 2008), statins (Morales‐Villegas, Di Sciascio, & Briguori, 2011), and P2Y_12_ receptor antagonists (Yang et al., 2013a,b). Others seem to block the protective effect of preconditioning through the inhibition of K_ATP_ channels (sulfonylureas, Cleveland, Meldrum, Cain, Banerjee, & Harken, 1997) by inhibiting A_1_R (aminophylline and bamiphylline) (Carr et al., 1997).

Researchers tried to translate these experimental findings with HBO into practice. In the case of skin transplantation in mice, HBO preconditioning was found to decrease the expression of adhesive molecules on T‐cell subsets, thus inhibiting the rejection of the allograft (Song, Sun, Zheng, & Zhang, 2010). In a model of adipocutaneous flap preparation in rats, HBO preconditioning was found to improve survival of the flap by attenuating the inflammatory response and increasing flap perfusion (Qi et al., 2013). Another study in rats showed that HBO preconditioning protected grafted skin flaps against subsequent I/R injury and improved skin flap survival rates, which was associated with the attenuation of inflammatory responses (Kang, Hai, Liang, Gao, & Liu, 2014).

Hundreds of trials have investigated HBO on various pathological conditions, mostly as a therapy. Only a few studies have investigated the preconditioning effects of HBO in humans (seven studies on the Cochrane Library to date). Sharifi et al. (2004) obtained favorable effects with HBO pretreatment in inhibiting restenosis after percutaneous coronary intervention in acute myocardial infarction. Alex et al. (2005) obtained favorable results on neuropsychometric dysfunction and inflammatory response after cardiopulmonary bypass with HBO treatment before on‐pump coronary artery bypass grafting (CABG). Yogaratnam et al. (2010) reported improved cardiac function and postoperative recovery in the case of HBO preconditioning before CABG. Jeysen et al. (2011) obtained HBO‐induced cardioprotection in a randomized clinical trial of 81 patients receiving CABG in relation to increased myocardial eNOS and HSP72. Li et al. (2011) conducted a randomized clinical trial of 49 patients receiving either on‐ or off‐pump CABG. The patients in the HBO‐preconditioned group had a significant decrease in S100B protein, neuron‐specific enolase, and troponin I (markers of cellular injury); a significant increase in catalase activity; decreased use of inotropic drugs; reduced length of stay; and better clinical outcomes compared to those in the control group. The off‐pump group showed no difference between HBO‐treated or control patients due to the missing ischemic period during the extracorporeal circulation.

Bosco et al. (2014) designed and conducted a clinical trial to verify the experimental findings to date in patients with pancreatic ductal adenocarcinoma who received a pancreaticoduodenectomy, but found no clear evidence upon modulation of HBO session on the studied cytokines.

## 9 SUMMARY

Experimental evidence suggests benefits of HBO when used as a preconditioning stimulus in the setting of I/R injury. Translating this into current practice requires the consideration of patients comorbidities and specific treatments in order to identify conditions that could blunt HBO benefits. In the case of pharmacological conditioning, the drug needs to be given in an adequate dosage and at specific moments to reach the threshold for protection. HBO seems to have certain advantages over drugs, not only because it can act on different complementary levels, but it also offers a reservoir of oxygen that may last for a few hours and may be of great importance in case of sudden hypoxia or ischemia, it improves endothelial function and rheology, and it decreases local inflammation and edema. Last but not least, oxygen reaches to the cellular level not only through being carried by blood, but also by diffusion from the interstitial tissue in which it reaches high concentration during HBO treatment, thus providing increased availability as compared to any drug. Moreover, the low cost and insignificant adverse events make HBO preferable to other types of conditioning strategies.

## CONFLICT OF INTEREST

None declared.
